# Editorial: Perception–Cognition Interface and Cross-Modal Experiences: Insights into Unified Consciousness

**DOI:** 10.3389/fpsyg.2016.01593

**Published:** 2016-11-22

**Authors:** Aleksandra Mroczko-Wąsowicz

**Affiliations:** Institute of Philosophy of Mind and Cognition, National Yang-Ming UniversityTaipei, Taiwan

**Keywords:** perception, cognition, concepts, cognitive penetrability of perception, cross-modal experience, multisensory integration, multimodal binding, the unity of consciousness

The present Research Topic explores closely related aspects of mental functioning, namely an interplay between perception and cognition, interactions among various sensory modalities, and finally, more or less unified conscious experiences arising in the context of these relations. Contributions emphasize a high flexibility observed in perception and may be seen as potential challenges to the traditional modular architecture of perceptual systems. Although the articles describe different phenomena, they follow one common theme—to investigate broadly understood unified experience—by studying either perception–cognition integration or the integration between sensory modalities. These integrative processes may well apply to subpersonal unconscious representations. However, the aim here is to approach phenomenal experience and thus a straightforward way of thinking about it is in terms of conscious perception.

One of the seemingly principled divisions in the human mind is between sense perception and high-level cognition. Traditionally, perception and cognition have been viewed as distinct, encapsulated domains operating independently of each other (Fodor, 1983, 1984, 2008; Pylyshyn, 1999, 2003; Barrett, 2005; Heck, 2007; Firestone and Scholl, 2014). However, recent studies support a different view about the impact of perception on cognition (Barsalou, 2009, 2012; Goldstone and Hendrickson, 2010; Prinz, 2011; Weiskopf, 2015) as well as the various ways in which perceptual experiences can be influenced by cognitive states such as thoughts, judgments, beliefs, intuitions, expectations, desires, mental images, and emotions (Brockmole et al., 2002; Raftopoulos, 2011; Lupyan, 2012; Macpherson, 2012, 2016; Siegel, 2012; Stokes, 2012; Bannert and Bartels, 2013; Vetter and Newen, 2014; Raftopoulos and Zeimbekis, 2016). Thus, although the mentioned division between perceiving and reasoning may seem conceptually clear and unambiguous, these mental domains become closely intertwined when our beliefs, expectations, or desires affect what we see, hear, or taste, leading to complex phenomenal states of a hybrid nature. No matter whether we assume that there is no dichotomy between perception and cognition (Clark, 2013; Lupyan, 2015) or instead assume that there is a principled difference and a joint in nature between perception and cognition (Block, Submitted; cf. Firestone and Scholl, 2015), cognitive-sensory interactions can and need to be accommodated within any of these accounts.

Perception has typically been studied in a single sense, mostly in the visual or auditory modality (Haynes et al., 2005; Gutschalk et al., 2008; Bekinschtein et al., 2009; Dehaene and Changeux, 2011; De Graaf et al., 2012). However, cross-modal experiences and heterogeneous multisensory interactions, in which input in one sensory modality elicits or modulates contents in another modality, reveal that such perceptual experiences cannot be easily categorized as belonging to only one of the senses (De Gelder and Bertelson, 2003; Stein, 2012). Furthermore, recent studies suggest examining the role of multisensory signals in perceptual consciousness (Chen and Spence, 2010; De Meo et al., 2015; Deroy et al., 2016). While processing sensory information in cross-modal cases is generally multisensory, the result of that processing can be interpreted as either just a sum of coexisting modality-specific representations or an intrinsically multisensory whole. Determining whether multisensory processing results in a decomposable conjunction of independent unisensory contents or in a multimodal holistic state that cannot be parceled out into modality-specific components would provide the needed characterization of the basic units of perceptual consciousness (Bayne, 2014). Still, it is important to realize that instances of successful multisensory integration and cross-modal binding facilitated by spatio-temporal or semantic congruence are not necessarily accompanied by unified experiences of objects across the senses and that the complex relationship between multisensory integration and perceptual consciousness remains to be clarified (Deroy, 2014; c.f. Spence and Bayne, 2015).

Recent years have seen a surge of novel interdisciplinary work questioning the received view of separate sensory systems and traditional conceptions of different mental domains operating independently (Shimojo and Shams, 2001; Driver and Noesselt, 2008; Bayne, 2010; Macpherson, 2011a,b; Mroczko-Wąsowicz, 2013, 2016; Bennett and Hill, 2014; Deroy et al., 2014; de Vignemont, 2014a,b; Mroczko-Wąsowicz and Nikolić, 2014; O'Callaghan, 2014; Matthen, 2015; Stokes et al., 2015). For instance, the occurrence of cross-domain interchange going beyond the link between the sense perception and the domain of abstract, conceptually represented entities, i.e., extending to the domains of bodily, motor, and emotional states provides challenges to standard methods individuating our epistemic abilities (O'Regan and Noë, 2001; Barsalou, 2008; Tajadura-Jiménez et al., 2011, 2015; Mroczko-Wąsowicz and Werning, 2012; De Coster et al., 2013; Weiss et al., 2013; Shapiro, 2014; Goldstone et al., 2015). Results of these studies point to a significant change in our understanding of perception; they demonstrate an emerging agreement on an integrative picture of perception incorporating informational interactions. All this indicates a need for a new research methodology. A full understanding of how the mind works requires considering multifaceted links holding between various mental domains and their mutual impact. Our mental faculties should not only be studied separately. They require a more holistic approach in order to uncover their extensive capacity for interactions producing differently unified conscious experiences.

Putting together scientific and philosophical concerns, this special issue encourages extending the study of perceptual experience beyond the single sense perception to advance our understanding of the complex interdependencies between different sensory modalities, other mental domains, and various kinds of unifying relations within conscious experience. It exhibits a remarkable need and benefit to study these phenomena in tangent, and so, the articles in this Research Topic examine a variety of ways in which our perceptual experiences may be cross-modal or multisensory, integrated, embodied, synesthetic, or affected by top-down influences.

Fulkerson argues that there are many forms of sensory interaction and unity, therefore classification of sensory systems and generated experiences is a matter of particular explanatory projects.

Connolly suggests that it is not an automatic feature binding mechanism that is responsible for our multimodal perceptual experiences, but rather an associative learning process that couples features from different sensory modalities so that we experience them as part of the same event.

Liang et al. investigate experiential ownership of bodily sensation and if it is guaranteed that a subject cannot be wrong about whether it is him who feels the sensation.

Van Leeuwen et al. propose several reasons for why the phenomenon of synesthesia and related alterations of brain networks and functional connectivity can be of merit for consciousness research.

Gray and Simner consider synesthesia and release phenomena in terms of disinhibited embodiment in sensory and motor systems respectively.

The following papers explore how perceptual processes can fail to be modular. They discuss a range of questions regarding cognitive effects on perception, including the issue of cognitive penetrability of perception.

In their contribution, Masrour et al. guide readers through philosophical issues of the debate on perceptual modularity, emphasizing results from cognitive neuroscience against the encapsulation thesis.

Marchi and Newen address the possibility of cognitive penetrability of perceptual experience in the domain of social cognition, namely visual experience of the facial expressions of emotion.

Nanay argues that the attribution of aesthetically relevant properties supervenes on one's perceptual experience, i.e., if there is a difference in such an attribution, there must also be a difference in perceptual experience.

Briscoe considers whether intentions for action penetrate visual experience of an object's size by analyzing various explanations of mechanisms possibly involved in such penetration.

Brown claims that theories of consciousness that see it as cognitive in nature or as an aspect of cognitive functioning such as the higher-order thought theory of consciousness provide a reasonable working hypothesis in the explanation of conscious experience.

There is also some grouping among the contributions discussing empirical results from their own studies on cross-modal associations, sensory integration, and unified conscious experience.

Brunel et al. demonstrate that cross-modal correspondences influence cross-modal integration during perceptual learning, leading to new learned units that have different stability over time.

Montoro et al. explore cross-modal metaphorical mapping of auditory emotion words onto vertical visual space and conclude that this association is not automatically activated but requires an explicit semantic evaluation of the emotion concepts to obtain an embodied effect.

Albertazzi et al. examine the existence of cross-modal associations between highly complex stimuli (i.e., materic painting and classical guitar music) due to patterns of qualitative similarity present in stimuli of different sensory modalities.

Finally, Winkielman et al. propose that unified consciousness is constructed from cross-modal inputs via integrated processing experiences, an experiential mechanism that combines signals of processing quality.

## Author contributions

The author confirms being the sole contributor of this work and approved it for publication.

### Conflict of interest statement

The author declares that the research was conducted in the absence of any commercial or financial relationships that could be construed as a potential conflict of interest.
